# Discovery of novel CSF biomarkers to predict progression in dementia using machine learning

**DOI:** 10.1038/s41598-023-33045-x

**Published:** 2023-04-21

**Authors:** Dea Gogishvili, Eleonora M. Vromen, Sascha Koppes-den Hertog, Afina W. Lemstra, Yolande A. L. Pijnenburg, Pieter Jelle Visser, Betty M. Tijms, Marta Del Campo, Sanne Abeln, Charlotte E. Teunissen, Lisa Vermunt

**Affiliations:** 1 Computer Science, Vrije Universiteit Amsterdam, Amsterdam, The Netherlands; 2 Alzheimer Center Amsterdam, Neurology, Vrije Universiteit Amsterdam, Amsterdam UMC location VUmc, Amsterdam, The Netherlands; 3 Amsterdam Neuroscience, Neurodegeneration, Amsterdam, The Netherlands; 4 Neurochemistry Laboratory, Department of Clinical Chemistry, Amsterdam Neuroscience, Vrije Universiteit Amsterdam, Amsterdam UMC, Amsterdam, The Netherlands; 5 Alzheimer Center Limburg, School for Mental Health and Neuroscience, Maastricht University, Maastricht, The Netherlands; 6 Department of Neurobiology, Care Sciences and Society, Division of Neurogeriatrics, Karolinska Institutet, Stockholm, Sweden; 7 Barcelonabeta Brain Research Center, Pasqual Maragall Foundation, Barcelona, Spain; 8 Departamento de Ciencias Farmacéuticas y de la Salud, Facultad de Farmacia, Universidad San Pablo-CEU, CEU Universities, Madrid, Spain; 9 CWI, Amsterdam , The Netherlands

**Keywords:** Machine learning, Prognostic markers

## Abstract

Providing an accurate prognosis for individual dementia patients remains a challenge since they greatly differ in rates of cognitive decline. In this study, we used machine learning techniques with the aim to identify cerebrospinal fluid (CSF) biomarkers that predict the rate of cognitive decline within dementia patients. First, longitudinal mini-mental state examination scores (MMSE) of 210 dementia patients were used to create fast and slow progression groups. Second, we trained random forest classifiers on CSF proteomic profiles and obtained a well-performing prediction model for the progression group (ROC–AUC = 0.82). As a third step, Shapley values and Gini feature importance measures were used to interpret the model performance and identify top biomarker candidates for predicting the rate of cognitive decline. Finally, we explored the potential for each of the 20 top candidates in internal sensitivity analyses. TNFRSF4 and TGF $\upbeta$-1 emerged as the top markers, being lower in fast-progressing patients compared to slow-progressing patients. Proteins of which a low concentration was associated with fast progression were enriched for cell signalling and immune response pathways. None of our top markers stood out as strong individual predictors of subsequent cognitive decline. This could be explained by small effect sizes per protein and biological heterogeneity among dementia patients. Taken together, this study presents a novel progression biomarker identification framework and protein leads for personalised prediction of cognitive decline in dementia.

## Introduction

Dementia is a clinical syndrome characterized by cognitive impairment, which progressively hampers activities of daily living. Dementia can be caused by various neurodegenerative diseases, such as Alzheimer’s disease (AD), Frontotemporal dementia (FTD) and Dementia with Lewy bodies (DLB)^1,2^. To allow future planning, providing an individualised prognosis about the disease progression after the diagnosis of dementia is important^3–5^. Advances in biofluid biomarkers have led to improved diagnostic tools for various underlying neurodegenerative diseases^6,7^. Nevertheless, the rate of disease progression is heterogeneous, even within the dementia type, and the lack of prognostic biofluid biomarkers remains a challenge^8,9^. While proteinopathies may differ among different types of dementia, shared biological processes may drive disease progression across various neurodegenerative diseases^10–13^. With novel discovery approaches and ultrasensitive assays, it is now possible to measure low-abundant protein markers in biofluids^7,14–17^. Such robust analytical techniques, combined with interpretable machine learning models provide the opportunity to develop biomarker panels to address the unmet need for good prognostic information for patients with dementia.

Cerebrospinal fluid (CSF) reflects the biological state of the brain and can provide valuable insights into the progression of dementia. Proteomics-based biomarker discovery using CSF is a promising approach to identify candidate proteins and biological pathways involved in the disease pathophysiology^14,17–24^. Proteomics studies often show subtle effects per marker, which combined contribute to a clear profile^14,25,26^. The patterns of up- and downregulated proteins can provide useful information about the mechanisms that might contribute to the disease progression, or provide protection against cognitive decline.

Reported values for an individual protein abundance and its alterations largely depend on the specific measurement technique used, which is a challenge in biomarker development^7,17,27^. While studies using traditional unbiased mass spectrometry techniques offer insights into the disease biology, it is challenging to detect low-abundant proteins and proceed to translation of biomarkers that can be used in clinical practice and treatment trials^7,28^. In this project, we used data obtained from highly sensitive and specific multiplex immunoassay-based proteomics technology^29^. As immunoassay-based techniques are commonly used in clinical practice, the use of this technology may facilitate translation of our findings to clinical settings^7,17,30^.

The objective of this study is to explore the potential of CSF proteins as biomarkers for predicting the speed of cognitive decline in individuals with dementia, while also investigating shared underlying mechanisms that contribute to cognitive decline rates. To achieve this, we employed supervised machine learning models, combined with traditional statistical techniques, which allows us to interpret combined effects to discover which markers in the CSF proteomics data of dementia patients predict the rate of cognitive decline. First, we defined slow- and fast-progressing groups based on the MMSE measurements. Second, we identified protein biomarker leads with predictive value based on feature importance analysis. As a final step, we explored the relationships between each of the top-ranked proteins and the rate of cognitive decline. Additionally, in order to understand the relationship between protein level abnormalities and dementia progression, we carried out functional enrichment and protein–protein interaction analysis.

## Results

### Defining fast and slow decliners

**Figure 1:**
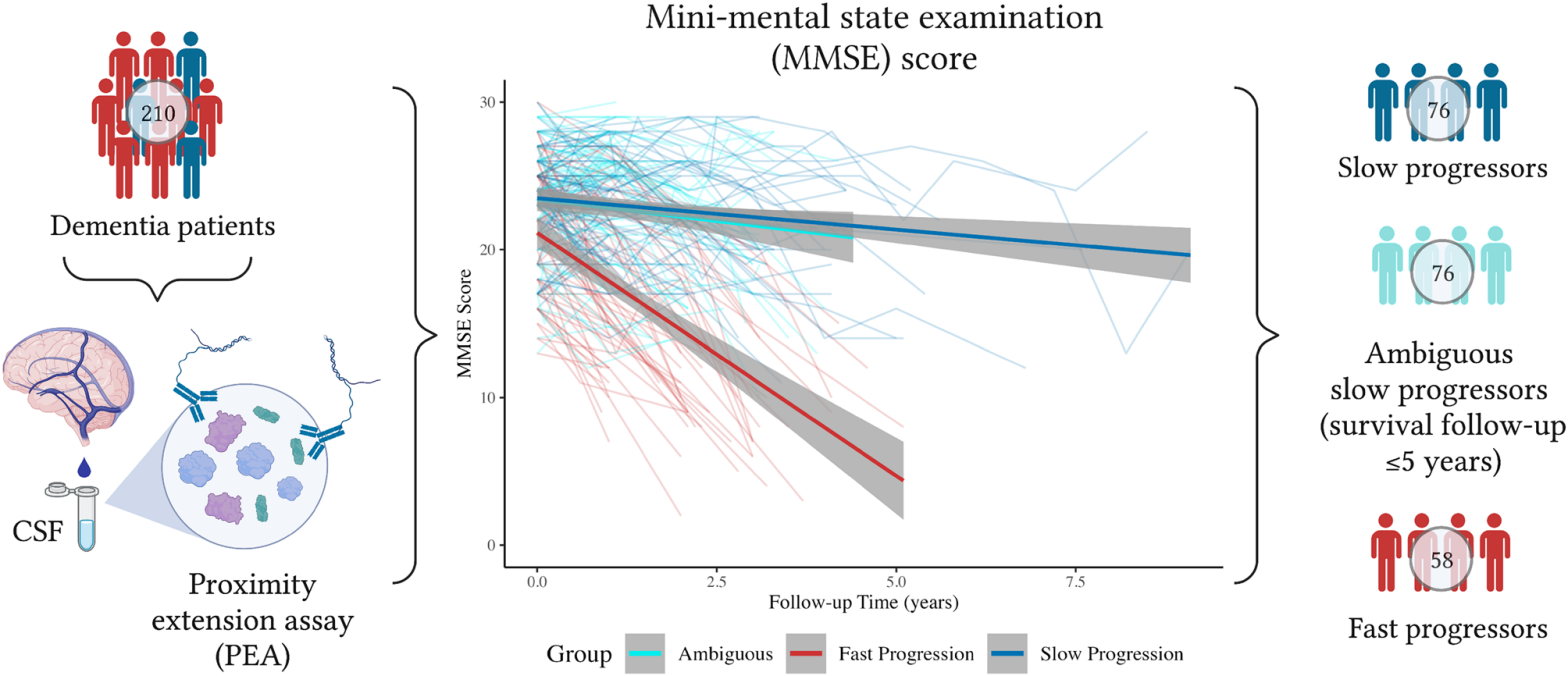
Patient demographics and identification of progressing groups. All CSF samples of patients were analysed by Olink proteomics and longitudinal MMSE measurements were collected. 210 patients with dementia and their MMSE scores over time were used to identify fast and slow decliners using latent class mixed models (LCMM). Shaded areas represent 95% confidence intervals. *CSF* cerebrospinal fluid, *MMSE* Mini-Mental State Examination.

We defined fast and slow decliners for the classification model based on their mini-mental state examination (MMSE) scores over time and survival follow-up (Fig. 1). The analysis included 210 dementia patients with an average of 3.3 MMSE observations over 2.3 years, who were grouped into two categories using latent class mixed models (LCMM)^31^ (Table 1). To ascertain a clear group contrast, 76 ambiguous patients in the slow decliner group with a survival follow-up $\le$ 5 years were excluded from the group comparison and machine learning classification analyses. The final slow-progressing group consisted of 76 patients with an average decline of 0.9 MMSE points per year (Table 1). The fast-progressing group consisted of 58 patients with an average decrease of 4.5 points on the MMSE per year and was younger than the slow-progressing group.

**Table 1:** Baseline demographics per group. *FU* follow-up, *MMSE* Mini-mental state examination, *CN* cognitively normal, *F* fast, *S* slow, *A* Ambiguous, *Comparison* group comparison statistics, *AD* Alzheimer’s disease, *DLB* dementia with Lewy bodies, *FTD* frontotemporal dementia, *CBD* corticobasal degeneration, *PSP* progressive supranuclear palsy, *CI* confidence interval, *n.s.* not significant, dash (–) not measured.

	Fast	Slow	Ambiguous	CN	Comparison
Sample size, n	58	76	76	196	–
Type of dementia, n, AD/DLB/FTD/CBD/PSP	43/8/4/2/1	38/21/13/2/2	38/18/6/6/8	–	n.s.
Age, years, average (SD)	62.6 (7.9)	66.7 (7.6)	68.4 (5.7)	58 (7.8)	F < S; F < A
Sex, male/female, n	38/20	50/26	55/21	123/73	n.s.
Education, years (SD)	12.2 (2.8)	11.9 (2.9)	10.8 (2.1)	12.1 (2.9)	A < F; A < CN
1st MMSE score, average (SD)	21.3 (4.3)	23.4 (3.3)	23.1 (3.8)	–	F < S; A < A
Decline in MMSE score per year (95% CI)	–4.5 (− 4.9; − 4.1)	− 0.9 (− 1.1; − 0.6)	− 0.9 (− 1.2; − 0.5)	–	S < F; A < F
n FU visits, average (SD)	3.1 (1.2)	3.8 (2.1)	3 (1)	–	F < S; A < S
Cognitive FU time, years, average (SD)	2.2 (1.1)	2.8 (1.9)	2 (1)	–	F < S; A < S
Survival FU time, years, average (SD)	5.3 (2.2)	7.2 (1.9)	3.6 (1.1)	–	F < S; A < S;
					A < F
Mortality, N (%)	35 (60)	38 (50)	51 (67)	–	n.s.

### Prediction of cognitive decline using machine learning

**Figure 2:**
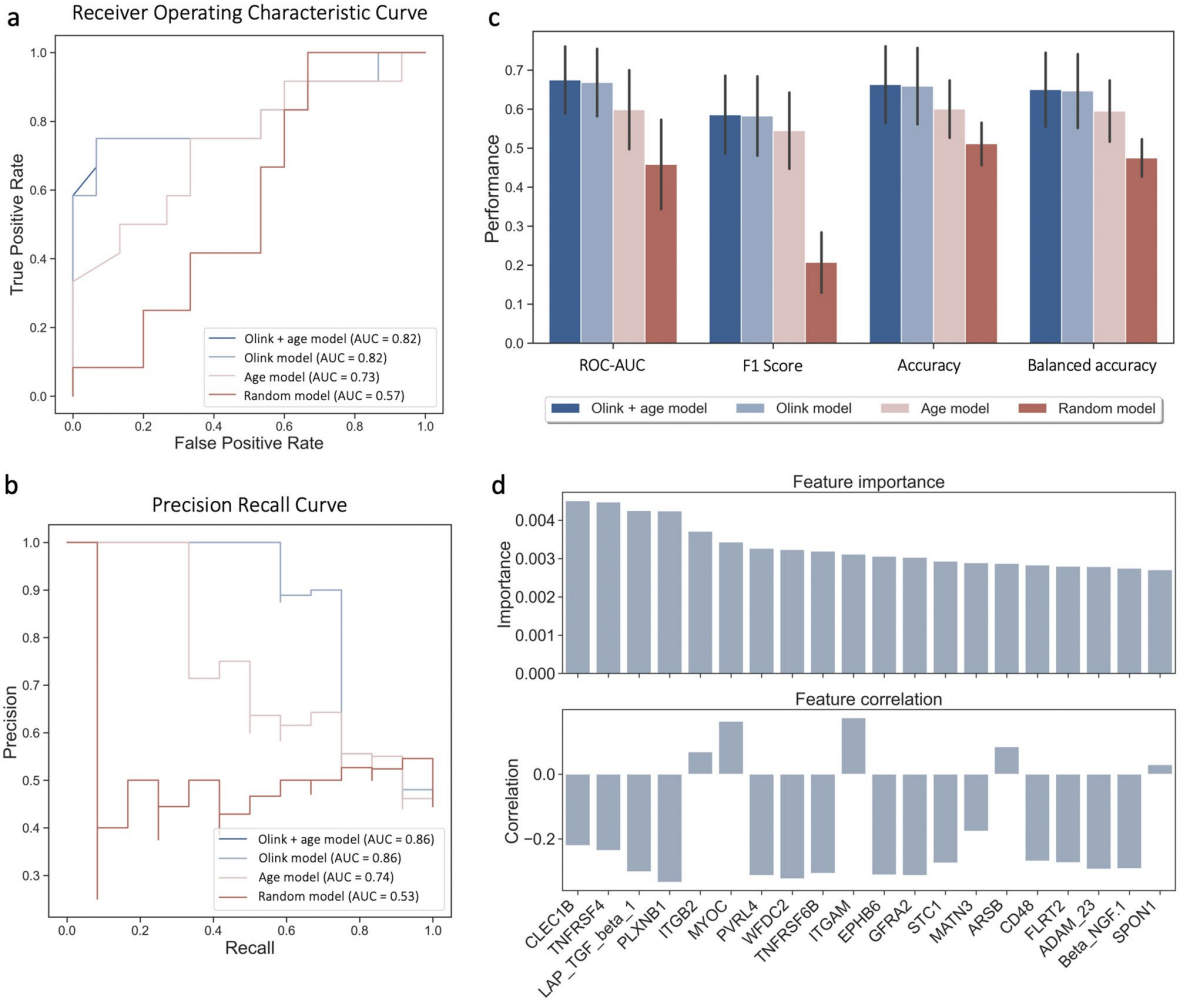
Performance evaluation of different RF classifiers. Four different RF classifiers were trained and evaluated on the independent test set. The Olink + age model was trained on all protein measurements and age; The Olink model is trained on 810 protein measurements. The age model is only trained on age; the Random model is trained on 810 protein measurements with shuffled labels. Four models were evaluated on different metrics: ROC–AUC, F1 score (F1), accuracy (ACC), and balanced accuracy (BACC). (**a**) Receiver operating characteristic and (**b**) precision-recall (PR) curves show the performance of the RF classifiers. The ROC–AUC and PR-AUC of the models using all the protein expression values achieve the highest values of 0.82 ad 0.86 respectively. (**c**) Different evaluation metrics for four models. Stratified train and test split were performed on 10 different random seeds. (**d**) Bar plot on top of the graph show top 20 most important features for predicting the rate of decline based on the Olink model. The bar plot below shows the correlation with the progression. Most of the biomarkers are negatively correlated, thus the lower abundance of these proteins in the CSF is associated with the faster decline in the MMSE trajectory. Protein relative abundance distributions of all selected biomarkers are shown in Fig. S1. The feature importance and correlation analysis for the Olink + age model are displayed in Fig. S2.

To assess which of the proteins contribute most to differentiating fast and slow decliners, a random forest (RF) classification model was trained on protein relative abundance values in CSF. The prediction task was the progression group. The performance of the RF classifier was evaluated on the held-out test set, which consisted of 20% of the labelled data (27 patients). Figure 2a–c displays the respective performance scores of four distinct RF classifiers that were trained and evaluated, namely: the Olink + age model, which was trained on all protein measurements and included age as a feature; the Olink model, which was trained on all protein measurements; the age model, which incorporated only age; and the Random model, which was trained on all protein measurements with shuffled labels. As the patient groups were imbalanced, various metrics, such as the area under the receiver operating characteristic curve (ROC–AUC), F1 score, accuracy, and balanced accuracy were employed to evaluate the models. The Olink model with and without age included as a feature demonstrated superior performance across all metrics with the ROC–AUC of 0.82. While adding age as a feature did not improve the performance, using age as a single feature (age model) resulted in an AUC of 0.73, which reflects that the fast decliners were on average younger than the slow decliners (see Table 1). Notably, using protein measurements results in a better classification model than only using age, and incorporating age as a feature results in a similar list of top biomarkers (17 overlapping proteins). All three models clearly outperform the random model, which was trained on shuffled labels.

**Figure 3:**
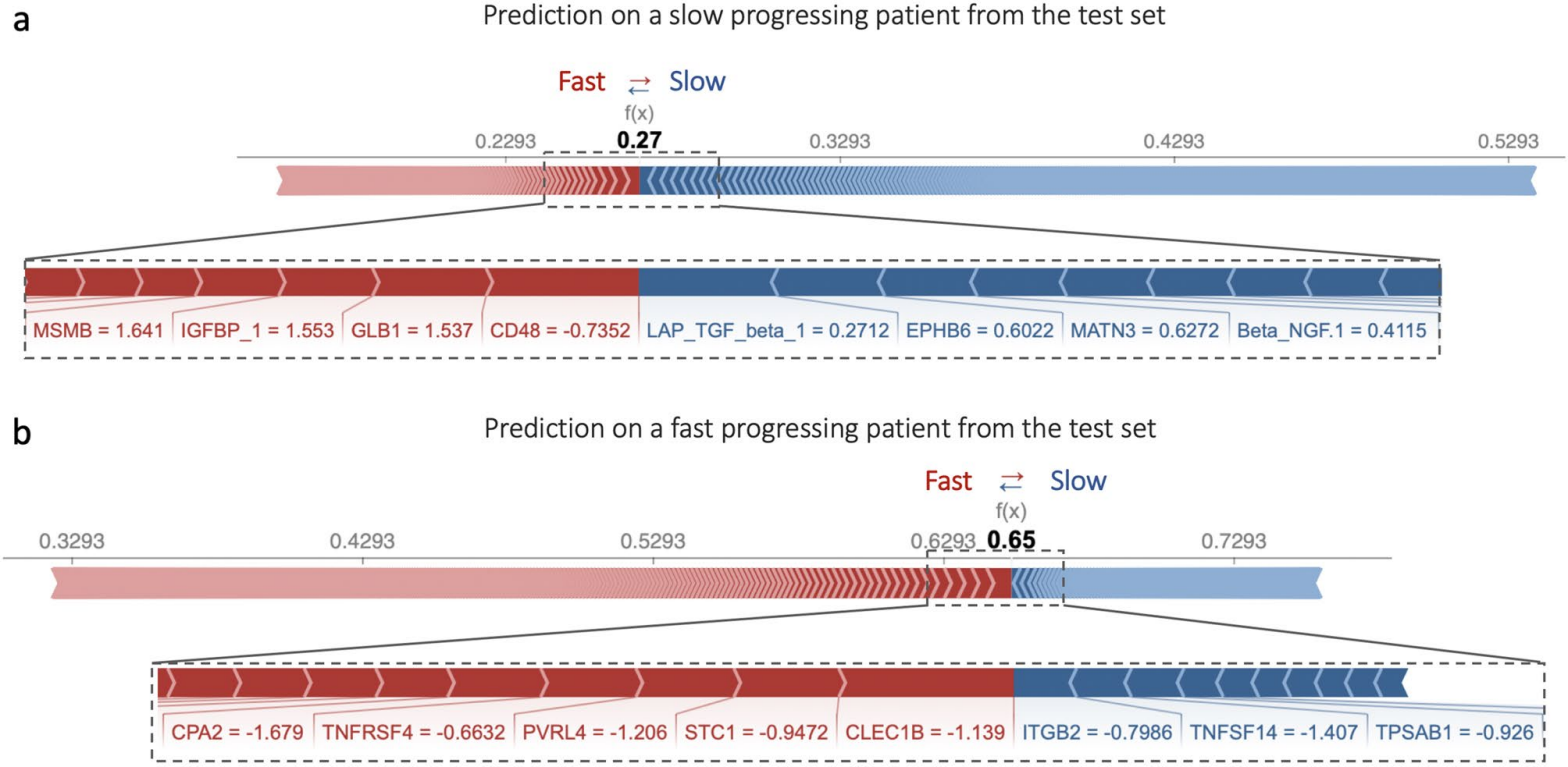
Shapley additive explanations (SHAP) analysis results for the model interpretability. For local interpretability figure shows two correctly classified patients from the test data in order to explain why each case receives its prediction and the contributions of these biomarkers. Note that the values indicated per biomarker are actual scaled values taken as input features by the prediction model. The model output values (0.27 and 0.65 respectively) are the predicted probability values for each observation (patient), which are not altered by the SHAP method. The width of the bar per feature corresponds to SHAP values indicating feature importance and the direction of the prediction. (**a**) The first patient received a score of 0.27, which is below the cut-off value of 0.5, and was thus classified as slow progressing. (**b**) Conversely, the second patient received a score of 0.65 and was classified as fast-progressing. Each observation (patient) gets its own set of SHAP values. Biomarkers in red contribute to the prediction being higher (Fast progressing, closer to 1), while features in blue push the value down towards the slow progressing group (0). SHAP summary plot shows us a birds-eye view of feature importance and how each biomarker drives the prediction (Fig. S3).

In order to identify biomarkers for subsequent cognitive decline, the Olink model (trained only on 810 protein measurements) was selected. Feature importance analysis was carried out in order to select biomarkers with the highest predictive value. Figure 2d shows the feature importance values and the direction of correlation of the top 20 proteins based on Gini feature importance, which included as top 3: C-type lectin domain family 1 member B (CLEC1B), Tumor necrosis factor receptor superfamily member 4 (TNFRSF4), and Transforming growth factor $\upbeta$-1 proprotein (TGF $\upbeta$-1).

To better interpret the model performance, Shapley additive explanations (SHAP) values were calculated (Fig. S3). Global SHAP value rankings differ from Gini importance analysis, but top proteins ranked with SHAP values largely overlap with the list of selected biomarkers based on Gini feature importance analysis. For local interpretability, we demonstrated two correctly classified patients from the test data (Fig. 3) to explain which biomarkers contributed to these predictions. SHAP analysis revealed several biomarkers that consistently demonstrated predictive value across multiple patients. For instance, patient A (Fig. 3a) showed elevated levels of TGF-$\upbeta$1, which was identified as a significant predictor for a slower cognitive decline. In another case (Fig. 3b), CLEC1B, TNFRSF4, and other biomarkers highlighted in red were found to be important contributors to the patient’s predicted fast progression. Notably, we observed a complex pattern of biomarker interactions for each patient, indicating that accurate prediction may require a multi-biomarker approach.

### Functional enrichment and protein–protein interaction analysis

To gain insight into the biological mechanisms that might be differentially affected in the fast and slow progressors, we conducted enrichment analyses. To adjust for age and sex, we assessed which protein expression values differed between the progression groups with nested linear models (Fig. S4)^32^. Ninety-two of the 95 proteins that showed significant differences between the fast and slow decliners (p-value $< 0.05$) showed negative effects, indicating that lower protein concentrations are associated with faster progression. In order to identify biological pathways and processes that are enriched with downregulated proteins in the CSF of fast-progressing patients, we performed enrichment analysis on these 92 biomarkers. Biological pathway analysis showed enrichment for GO terms and KEGG pathways associated with cell adhesion, cell signaling, and immune response pathways (Fig. S5). Note that using the entire human genome as a background is typically used for functional enrichment analysis for *unbiased* proteomics. Here it enabled us to identify potentially interesting pathways that could be associated with the dementia progression based on our results. To check the potential bias introduced by selecting proteins for the Olink panels, we repeated the analysis with defined background of all biomarkers used in our study and found that KEGG pathways connected to axon guidance (p-value = 0.01), TGF-$\upbeta$ signalling (p-value = 0.05), cytokine–cytokine receptor interaction (p-value = 0.09), and MAPK signalling pathway (p-value = 0.09) showed an enrichment. Signalling receptor activity (p-value = 0.02), and molecular transducer activity (p-value = 0.02) showed significant enrichment in GO terms. Additionally, we explored protein–protein interaction networks for three most promising proteins based on feature importance analysis using a graph-based approach of the STRING database^33^. Our analysis revealed a strong interaction between CLEC1B and Podoplanin (PDPN), with CLEC1B acting as a receptor for PDPN (Fig. S6). Moreover, PDPN is a shared physical connection between TNFSF4 and CLEC1B. It is conceivable that this interaction plays a role in cell migration and adhesion, which also appears in the enriched GO terms (Fig. S5). Additionally, TNFRSF4, a costimulatory molecule implicated in long-term T-cell immunity, was found to primarily interact with other tumor necrosis family members, as well as chemokine receptor CXCR4 and T-cell-specific surface glycoprotein CD28, indicating involvement in T-cell activation. Furthermore, TGF-$\upbeta$-1 was observed to interact with a range of proteins beyond those involved in the TGF-$\upbeta$ signaling pathway and its receptors, such as Endoglin, Decorin, and Matrix metalloproteinase-9, as well as Interleukin-6, suggesting potential roles in fibril formation and extracellular matrix organisation.

### Internal sensitivity and external validation

**Figure 4:**
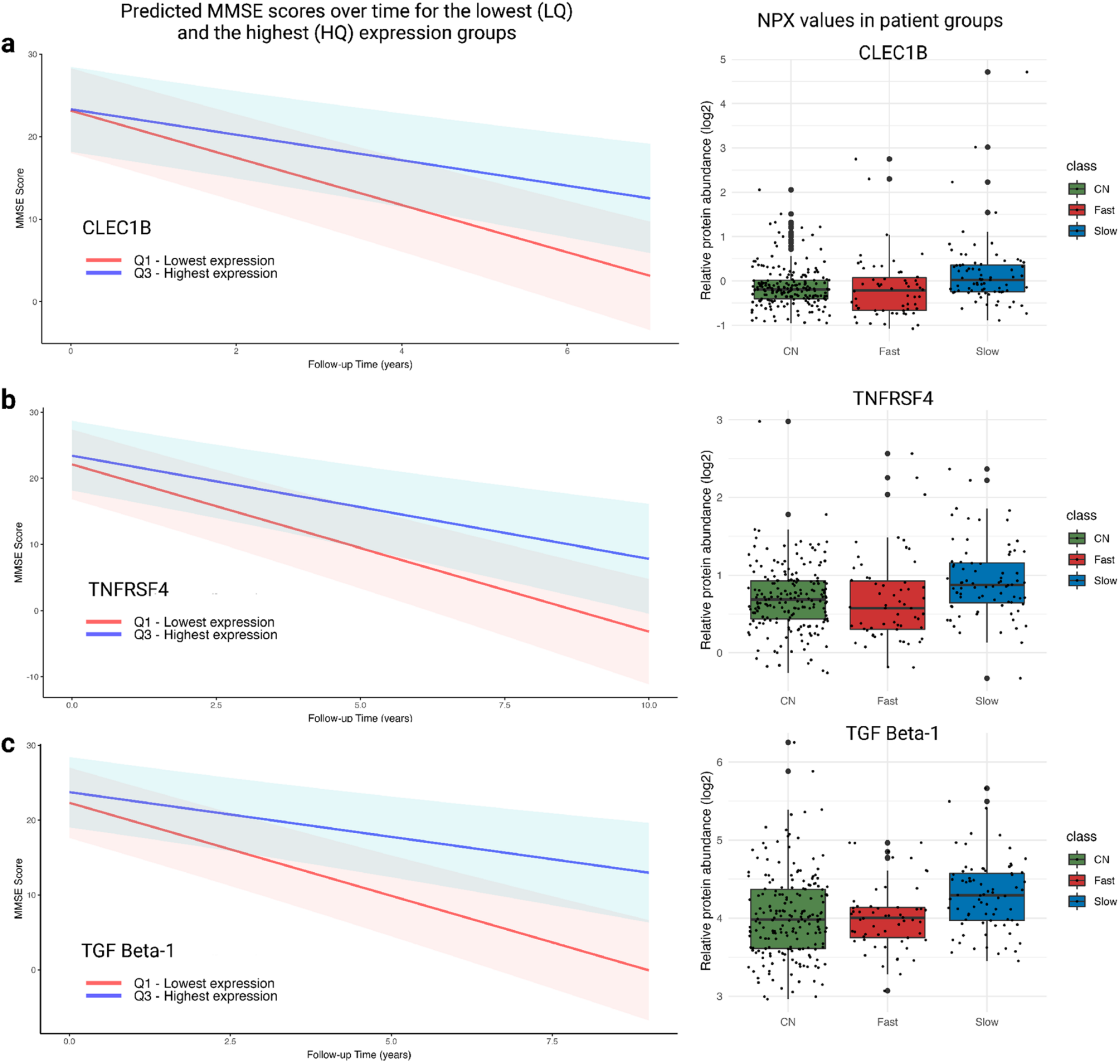
Internal sensitivity analysis of top 3 protein leads based on feature importance and SHAP values. On the left side figure shows predicted MMSE scores over time in the lowest and the highest expression quartiles based on CLEC1B (**a**), TNFRSF4 (**b**) and TGF $\upbeta$-1 (**c**). Shaded areas represent confidence intervals of 95%. The right figures show normalised protein expression (NPX) values for CLEC1B (**a**), TNFRSF4 (**b**) and TGF $\upbeta$-1 (**c**) in cognitively normal individuals (CN), fast and slow progressors respectively. NPX distributions of all selected biomarkers are depicted in Fig. S1.

As a final step (Fig. 2d), we were interested in the ability of individual biomarkers on our list of 20 selected candidates to predict subsequent cognitive disease. To get insight into the association with a cognitive decline for each individual protein, we took the reversed approach and grouped all dementia patients (n = 210) based on the expression levels of our biomarker leads. For each protein, we selected patients with the lowest (LQ) and the highest (HQ) expression values and assessed the association with cognitive decline (LQ-HQ). Eleven out of 20 top markers showed a significant difference (Table S1, Fig. 4). Additionally, to test the effect of reducing the heterogeneity, we analysed only patients with AD dementia (n = 119), which resulted in a stronger contrast between the two groups (Table S1). Figure 4 also depicts the relative abundance values for the individual biomarker across cognitively normal, fast, and slow-progressing patient groups. Protein levels were elevated in slow-progressing patients, but they show in fast progressing patients similar levels to cognitively normal controls (Table 1).

For validating the potential of individual biomarker candidates externally, we explored our biomarker leads in individuals with dementia from the publicly accessible ADNI cohort (https://adni.loni.usc.edu), using the same approach. Only three proteins of the 20 proteins had been measured in ADNI: of these $\upbeta$-NGF-1 and TGF $\upbeta$-1 showed the same direction, namely, lower levels were associated with faster progression over time (not significant). On the other hand, SPON1 showed significant differences between the lowest and the highest expression quartiles in the opposite direction compared to the ADC cohort, being downregulated in fast-progressing patients (Table S1).

## Discussion

In this research, we aimed to discover CSF protein biomarkers and biological mechanisms predictive of rapid decline in individuals with dementia. To disentangle the heterogeneous disease trajectory, patients’ MMSE scores over time in combination with survival data were used to identify two groups with a rapid and slow decline. To find proteins associated with steeper decline, the expression values of 810 proteins, measured with PEA proteomics technology were used to train RF classification models, and select the top 20 biomarker candidates based on Gini feature importance. Eleven of the top 20 CSF biomarker leads associated with the rate of cognitive decline in patients diagnosed with dementia are involved in signalling pathways (TNFRSF4, TGF $\upbeta$-1, CLEC1B, GFRA2, TNFRSF6B, EPHB6, PLXNB1), cell migration (EPHB6), and cell adhesion (PVRL4, EPHB6). There are several implications of these findings. We found that applying a machine learning algorithm on a multidimensional biomarker dataset has the potential to identify novel markers that could aid in a personalized prognosis. At the same time, predicting the disease progression in dementia was not a trivial task and none of our top markers stood out as strong individual predictors. This might be explained by the biological heterogeneity among dementia patients^34^. The effect sizes per protein seem to be small, and proteins might have a good predictive value in a subgroup of the patients or only in combination with other markers. The CSF biomarker leads selected in this study provide a novel basis for validation studies to reach the ultimate aim to provide better prognostic information for a clinical setting. Second, such biomarkers could have an interesting application for clinical trials, as the sample size required for a trial could be reduced by enriching with participants who are more likely to decline faster^35^.

The pattern of differences in protein levels between slow and fast decliners enriches our perspective on dementia progression (Fig. S5). Our findings suggest an impairment of processes connected to cell signalling and immune response. Immune response pathways have been reported to be dysregulated based on the meta-analysis of AD proteome from post-mortem studies^27^. Pathways that are typically upregulated in neurodegenerative diseases, such as cytokine-mediated signalling, indicating the presence of neuroinflammation^27,36,37^, were downregulated in fast decliners with dementia (Fig. S1). The upregulation in slow decliners could indicate the protective functions of a pathway. Another reason for the lower levels in fast-progressing patients could be that this reflects more neuronal dysfunction or neuronal loss as a result of the preceding neurodegeneration^38,39^. To determine whether the observed upregulation is a compensatory effect or a protective pathway, as well as to investigate whether certain proteins reflect an inherent protective trait, longitudinal measures within individuals are necessary. Additionally, performing pQTL analyses on the genetic traits could provide further insight into these questions. It may also be useful to explore whether markers decrease as the disease progresses.

CSF proteomics data has been previously used to assess proteomics signatures for conversion from MCI to AD dementia^26^. Several biomarkers from the list of our biomarker leads, namely, TNFRSF4, MATN3, and $\upbeta$-NGF-1 were also downregulated in the pre-dementia disease stage. $\upbeta$-NGF-1 also showed this same direction in the ADNI dataset. Another study also showed that for TGF $\upbeta$-1 decreased levels were associated with a higher probability of progressing to AD dementia in MCI patients^22^. In that study, however, increased, instead of decreased CSF levels of CLEC1B associated with progression to dementia. In serum, increased levels of TGF $\upbeta$-1 have been associated with AD incidence^40^, which may be in line with a recent study in CSF and plasma indicating that the direction of protein level abnormality tends to be opposite between these matrices^41^. Plasma proteomics focussed on inflammation and vascular injury have been associated with cognitive decline^16,42^, but there was no overlap with our top biomarkers in CSF. Although our study identified promising biomarkers, it is important to note that there is a scarcity of research on the potential role of these biomarkers in the disease progression of non-AD dementias.

Two proteins ranked highest based on feature importance analysis, SHAP and consistent findings in the literature are TNFRSF4 and TGF $\upbeta$-1. While little is known about TNFRSF4, other than that it is involved in neuronal cell signalling pathways, the TNF-$\alpha$ receptor signalling pathways, namely TNFR1 and TNFR2 have been investigated in neurodegenerative diseases^43^. A study carried out in triple-transgenic AD mice (3xTg-AD) reported that deletion of both TNFR1 and TNFR2 significantly worsened AD pathology^44^. TNF-$\alpha$ was shown to have divergent roles in neurodegenerative disorders, including neurodegenerative and neuroprotective effects, which appear to depend on its signalling via the family of TNFR family members^45^. Based on our results TNFRSF4 may exert protective effects, as lower CSF levels are correlated with faster progression.

Another biomarker lead of interest is TGF $\upbeta$-1, feature importance and SHAP analysis combined with the functional enrichment results pointed out TGF $\upbeta$-1 as one of the most interesting. TGF $\upbeta$-1 is a multifunctional protein, a neurotrophic factor that regulates the growth and differentiation of various cell types^46^. TGF $\upbeta$-1 is involved in various processes, including immune response, microglia function, and homeostasis^47^. In previous work, TGF $\upbeta$-1 was proposed to have neuroprotective effects against Amyloid-$\upbeta$-induced neurodegeneration^48,49^. An earlier study investigated aged AD mice and reported a 50% reduction of Amyloid-$\upbeta$ load with a modest increase in astroglial TGF $\upbeta$-1 production^50^. A recent multi-platform proteomic co-expression analysis of AD in CSF identified strong signals with TGF $\upbeta$ signalling pathway^41^. Lower levels of TGF $\upbeta$-1 in CSF of fast decliners compared to the slow decliners suggests that lower levels of TGF $\upbeta$-1 might reflect a lack of neuroprotective effects of TGF $\upbeta$-1.

One of the strengths of the framework is that we integrated high-scale low abundant proteomics to address the difficult challenge of assessing disease progression in dementia. The proteomics approach gives us insights into proteins that would never have been considered or even found before. While the number of proteins is still limited by the availability of antibodies, it is due to the antibody-based technology that translation to a panel or single assays could be feasible^17^. However, the validation of our findings, especially across different proteomics methods is challenging, since the measurements of protein abundance depend on the nature of the techniques^41^. Olink proteomics measurements are based on antibody binding, while mass spectrometry analysis provides peptide counts. Therefore measurements are challenging to compare as the surface accessibility of a protein can be influenced by various post-translational modifications causing the detection of two different variants or fragments of a biomarker^51,52^. Another novel part of this framework is the use of data-driven models to find proteins that predict a rapid decline.

Nonetheless, the study also had limitations. Although LCMM is especially useful for heterogeneous populations^31^, defining fast and slow decliners is a crude clustering step. It should be kept in mind that disease progression speed is a continuum, but the labels referring to fast and slow-progressing groups were required to allow training of the machine learning classifiers. In line with our validation procedures, the next step needed is to assess the association of selected protein biomarkers with the rate of decline and clinical milestones.

A further constraint is the choice of cognitive tests used to classify patients. MMSE is a well-known cognitive assessment tool that has been extensively validated and is popular due to its ease of administration, particularly for elderly or severely cognitively impaired individuals, and can be used as a progression-tracking tool^53,54^. Nevertheless, the MMSE is susceptible to external factors that can compromise its accuracy, including age, education level, and medical conditions^55^. Future studies might consider using alternative tests specifically developed and validated for detecting cognitive decline over time, such as the RBANS or Cognitive-Functional Composite (CFC)^56–60^. A composite, such as CFC, can combine measures of cognitive function and functional ability, providing a more comprehensive assessment changes in a patient’s abilities than the MMSE.

Another limitation of our study was the naturalistic clinical follow-up, which is why we excluded an ambiguous group from the initial machine learning classification analysis in order to obtain a clearer distinction between fast and slow decliners. This highlights the value of collecting disease progression follow-up data until close to mortality.

ML models can be susceptible to bias and overfitting, which can impact the generalisability of the models. To address these issues, first, we used a diverse cohort of dementia patients to increase the generalisability of our findings. Selected subsets of biomarkers with high predictive power were additionally tested using an internal sensitivity analysis. In order to prevent overfitting, we used a relatively simple model architecture and a held-out validation approach with multiple random seeds to ensure that the split was representative of the population. While cross-validation provides a more robust estimate of the model’s performance, held-out validation was preferred due to the size of the dataset.

We also need to acknowledge that age difference between the fast and slow progression groups could potentially impact the proteomics biomarker discovery. As the goal was to establish biological measures of disease progression, we tested the effect of age in our models to assure that the proteomics measures could not be replaced by age only. Our results indicated that the biomarker measurements had an additive effect. Moreover, including age in the biomarker model did not affect the selection of top biomarker candidates, which is an indication that these biomarkers indeed contribute to capturing a process involved in the speed of progression.

While the use of CSF provides a more precise reflection of the CNS’s biochemical processes than blood, the invasive nature of collecting it presents a major constraint to its broad implementation. Blood, on the other hand, is a less invasive alternative that can be obtained frequently. However, searching for biomarkers in plasma has its disadvantage as the concentrations of CNS-related proteins tend to be low, and the protein levels in plasma can be influenced by all organs and cells in the body, which makes it challenging to identify CNS-specific changes.

The inclusion of multiple dementia types was required to maintain a sufficiently large dataset for the analysis and pick up small differences which increase the heterogeneity. Importantly, not all dementia types decline similarly in all cognitive domains, which might have affected the decline in MMSE scores, which assesses global cognition^61,62^. Despite differences in clinical presentation and neuropathological hallmarks, there is evidence of shared mechanisms across different types of dementia, that we could detect in our study. For instance, chronic neuroinflammation is known to be present in AD, DLB, and FTD^10,11,13^, or lysosomal dysfunction, another mechanism implicated in both FTD and DLB pathophysiology^63,64^. Given that the results remain consistent within the AD group alone, it is improbable that the heterogeneity from non-AD dementia’s accounts for the findings. However, a limitation of the inclusion of multiple dementia subtypes in a single study is that it may mask the disease-specific effects, and it is crucial to obtain replication data to understand the role of each marker within specific disease groups. We did find that the significant contrast between the lowest and the highest quartiles became stronger when analysing only the AD dementia patients. This could indicate that some protein changes are more specific for AD and can be overshadowed by combining all dementia types. Therefore, larger patient cohorts for individual dementia types can potentially provide more insights into the disease progression. Lastly, we used cross-sectional proteomics data, and with longitudinal proteomics measurements, the intra-individual dynamics of the protein levels can be assessed in the future.

In summary, we identified several candidate CSF protein leads that might carry prognostic value and can potentially help predict the speed of the disease progression in dementia patients. Proteins showing a negative correlation with fast progression are enriched for cell adhesion, cell signalling, and immune response pathways, and might indicate the lack of a protective response in these patients. Together, these results suggest that a CSF biomarker panel following future validations can potentially offer useful prognostic information.

## Methods

### Study design and participants

The patients were part of the Amsterdam Dementia Cohort (ADC^5^), the memory clinic cohort from the Alzheimer Center at the Amsterdam UMC. On their first visit to the center, patients received a full diagnostic work-up, including a clinical and neuropsychological evaluation, magnetic resonance imaging (MRI), and a lumbar puncture. Patients are followed annually with clinical and neuropsychological evaluations. The local Medical Ethical Committee gave approval and the patients gave written consent for the use and storage of the clinical data and biomaterial for research purposes and biobanking^5^. We selected dementia patients that had the CSF proteome measurement, at least one follow-up visit (after $> 6$ months) with Mini-Mental State Examination (MMSE) score and survival data available. We also selected a control group of patients with the CSF proteome measurement, a normal CSF AD biomarker profile, and a diagnosis of subjective cognitive decline, confirmed by normal neuropsychological test scores. The final dataset consisted of 210 individuals with dementia and 196 cognitively normal (CN) individuals. Individuals with dementia were diagnosed with Alzheimer’s disease dementia ($\hbox {n}=119$), dementia with Lewy bodies ($\hbox {n}=47$), frontotemporal dementia ($\hbox {n}=23$), corticobasal degeneration ($\hbox {n}=10$), or progressive supranuclear palsy ($\hbox {n}=11$).

### Protein measurements

All CSF samples have been analyzed by Olink Proteomics (“Olink Proteomics,” 2021) and harmonised between batches to account for possible batch effects as described in^17^. Briefly, 979 proteins were measured with 11 Olink Target 96 multiplex panels based on the Proximity Extension Assay (PEA) technology. This technology employs matched antibodies, with strands of DNA attached to them, that bind to proteins in the CSF resulting in the hybridization and extension of these DNA strands. This creates a unique barcode for each protein, which is then amplified using qPCR. The amount of amplified DNA is translated back to the amount of protein in the samples. Olink Proteomics returns the protein expression using the normalized protein expression, a log2 scale unit for relative quantification. For all proteins, the lower limit of detection (LOD) was set at three standard deviations above background expression. The background was defined as the median expression of the negative controls on that specific plate. A report on the performance of each of the assays on the multiplex panels can be found on the manufacturer’s website (https://www.olink.com).

### Statistical analysis and machine learning

Statistical analysis was performed using Python version 3.9.7 and R version 4.0.3. Comparisons of baseline characteristics between patient groups were performed with one-way ANOVA, Kenward-roger, Kruskal tests and Chi-squared tests when appropriate. If significant differences were found, we performed post-hoc comparisons with Tukey’s tests p-values adjusted for multiple comparisons with the Hochberg procedure.

#### Data curation

Proteins with an expression value above the limit of detection (LOD) of 10% were maintained. The final dataset contained 810 assays (781 unique proteins). There were five missing data points and three individuals had one missing panel due to technical errors, which were imputed according to the multivariate normal distribution using the MICE package (0.001%)^65^. Out of the 406 patients, four dementia patients did not have an MMSE score at baseline. These MMSE scores were imputed according to the multivariate normal distribution with the MICE package^65^.

#### Identifying progression groups

In order to identify patient groups with comparable rates of cognitive decline, latent class mixed models (LCMMs) were fit using R package lcmm adjusted for dementia type^31^. Progression was represented by the MMSE scores over time. LCMMs compute latent groups that hold subjects with comparable progression trajectories. In order to create groups with subjects that had comparable progression slopes, a random intercept, and a fixed slope were applied. As a result, the model that contained two groups with the lowest Bayesian information criterion (BIC) was selected.

Importantly, some trajectories that were classified as slow had a short follow-up of survival. Thus, there is not enough follow-up data to assess the status correctly. Consequently, patients classified as slow progressors but with survival $\le 5$ years were removed and labelled as ambiguous. The final labelled dataset contains four groups: fast progressors ($\hbox {n}=58$), slow progressors (76), and ambiguous, (slow progressors with fast mortality, $\hbox {n}=76$). The ambiguous group was not used to train machine learning classifiers and the group comparison. The ambiguous group ($\hbox {n}=76$) was included in the internal sensitivity analysis to evaluate individual biomarkers. The cognitively normal individuals were only used as a reference for visualisation in Fig. 4 and Fig. S1.

#### Machine learning

We used a supervised machine learning algorithm random forest, an ensemble learning method using a multitude of decision trees. Protein relative abundances in CSF were used as features and the target variable is referred to as the progression group. Protein measurements were transformed with robust scaling. The curated dataset contained 134 patients and 810 protein measurements. Data were split into 80% for training and 20% for testing. Since our classes were not balanced, this split was stratified and performed 10 times with different random seeds (over 10 iterations). 4 different Random forest classifiers were trained on 80% of the data and evaluated on 20% of the held-out test set. The Olink + age model is trained on all protein measurements and age. The Olink model is trained only on 810 protein measurements. The age model is only trained on age. The random model is trained on 810 protein measurements with shuffled (wrong) labels. Four models were evaluated on different metrics: area under the receiver operating characteristics curve (ROC–AUC), F1 score, accuracy, and balanced accuracy. The Olink model (only trained on protein measurements) was selected to identify biomarkers with the biggest predictive value. The specifics of the Olink model chosen for downstream analysis were as follows: random state = 0, n estimators = 10,000, max features = 10. Feature importance was determined using Gini importance analysis, which provides a ranking and the importance score for each feature (biomarker). Gini importance analysis does not provide an effective direction.

In order to understand the effect of the most important proteins, pairwise correlation analysis was applied and the correlation between each feature and the progression group was calculated. Shapley Additive explanations (SHAP) analysis was carried out to further interpret the predictions. SHAP values are a widely used approach from cooperative game theory. SHAP values explain the difference between the average and the actual model prediction^66^. The collective SHAP values show how each biomarker contributes, either positively or negatively, to the target variable, in our case the progression group. SHAP summary plot creates numeric measures to see which features are important to a model, providing rather a birds-eye perspective on feature importance. A higher SHAP ranking could mean a large effect for a few predictions, but little effect overall, or a medium effect for all predictions. It is also possible to calculate ‘feature interactions’ for exploring various feature combinations that are used together to make predictions. Here we calculated SHAP values for both, global and local interpretability. For local interpretability, SHAP values are computed for individual patients, with values approaching zero indicating the model’s high confidence that the patient belongs to the slow-progressing group, and vice versa. This approach allows us to identify the specific impact of each biomarker on the model’s decisions, including both accurate and inaccurate predictions.

For the classification task, all metrics are derived from true and false positives and true and false negatives (together referred to as the *confusion matrix*). Positive refers to fast progressors and negative—to slow progressors. True positives (TP) are correct predictions of fast-progressing patients, and false positives (FP) are slow-progression cases that are incorrectly predicted to be fast-progressive. True negatives (TN) are correctly predicted negative or slow progression cases, and false negatives (FN) are fast progression cases that the machine learning method predicts incorrectly. The following evaluation metrics were used to assess the performance of the RF classifiers.

1. *Specificity* (spec) or *True Negative Rate* (tnr)   $= \, \textsc {tn}\,/\,(\textsc {tn}+\textsc {fp})$
2. *Error*, *False Positive Rate* (fpr)   $= \, 1-$Specificity   $= \, \textsc {fp}\,/\,(\textsc {tn}+\textsc {fp})$
3. *Sensitivity* (sens), *Recall*, *Coverage* or *True Positive Rate* (tpr)   $= \, \textsc {tp}\,/\,(\textsc {tp}+\textsc {fn})$
4. *Accuracy* (acc)   $$= \, (\textsc {tp}+\textsc {tn})\,/\,(\textsc {tp}+\textsc {fn}+\textsc {tn}+\textsc {fp})$$
5. *Balanced accuracy* (bacc)   $= \, (\textsc {tpr} + \textsc {tnr})\,/\,2$
6. *Precision* (prec) or *Positive Predictive Value* (ppv)   $= \, \textsc {tp}\,/\,(\textsc {tp}+\textsc {fp})$
7. F1   $$=$$   $$2\,\times \textsc {prec} \times \textsc {sens} \,/\, (\textsc {prec} + \textsc {sens})$$
8. AUC-ROC: area under the ROC curve (Sensitivity vs. $1-$Specificity)
9. AUC-PR: area under the Precision/Recall (P/R) curve

### Validating the top 20 promising biomarkers

#### Assessment of individual biomarkers—internal sensitivity

Having established the list of 20 CSF proteins with the highest predictive value, we moved on to explore the MMSE trajectories without labelling by including all dementia patients (n = 210). For each biomarker, we selected individuals with the lowest and the highest quartile (LQ, HQ) based on the relative abundance of a respective biomarker. Linear mixed-effects models using lme4 package^67^ were fit to for each of the top 20 biomarkers to predict MMSE trajectories over time in high and low expression groups (LQ-HQ).

#### External validation

For the external validation and the relevance of selected biomarkers, we explored the relationship of these markers with MMSE trajectories in AD dementias with MRM MS Spectrometry CSF measurements in the ADNI dataset (see https://www.adni-info.org). Data used in the preparation of this article were obtained from the Alzheimer’s Disease Neuroimaging Initiative (ADNI) database (adni.loni.usc.edu). The ADNI was launched in 2003 as a public-private partnership, led by Principal Investigator Michael W. Weiner, MD. The primary goal of ADNI has been to test whether serial magnetic resonance imaging (MRI), positron emission tomography (PET), other biological markers, and clinical and neuropsychological assessment can be combined to measure the progression of mild cognitive impairment (MCI) and early Alzheimer’s disease (AD). Three of our top candidates were also available in the ADNI dataset. We fitted the same mixed-effects models for the internal sensitivity analyses to predict MMSE trajectories over time. High and low-expression quartile groups of patients were defined based on individual biomarkers. The LQ-HQ trajectories and the trend of the effect were compared with our results.

### Functional analysis

In order to identify biological pathways and processes enriched with the proteins associated with dementia progression, we selected proteins that were significantly different, $\hbox {p}<0.05$, between fast and slow-progressing groups based on nested linear models adjusted for age and sex. These results were merged with the machine learning analysis results, selecting biomarkers with non-zero feature importance values resulting in 95 proteins. Only 3 proteins (ITGAM, MYOC, and CAMKK1) were upregulated in the fast progressors, which were removed to only focus on downregulated proteins and their enrichment. In total 92 proteins were used for the functional analysis using Bioconductor (Release 3.15) tool ClusterProfiler, a universal enrichment tool for interpreting omics data^68^. Initially, the functional enrichment analysis was performed without defining a background set of genes. Since Olink panels represent pre-selected sets of proteins, we performed the same analysis with the defined background of the 810 proteins in our study.

## Supplementary Information

Supplementary Information.

## Data Availability

Deidentified data and code related to this work can be obtained by request for purposes of replicating results from the corresponding author.

## References

[1] Livingston G (2020). Dementia prevention, intervention, and care: 2020 report of the lancet commission. Lancet.

[2] Schwarzinger M, Dufouil C (2022). Forecasting the prevalence of dementia. Lancet Public Health.

[3] Boustani MA (2011). Implementing innovative models of dementia care: The healthy aging brain center. Aging Mental Health.

[4] Rhodius-Meester HF (2018). Disease-related determinants are associated with mortality in dementia due to Alzheimer’s disease. Alzheimer’s Res. Ther..

[5] Van Der Flier WM, Scheltens P (2018). Amsterdam dementia cohort: Performing research to optimize care. J. Alzheimer’s Dis..

[6] Hansson O (2021). Biomarkers for neurodegenerative diseases. Nat. Med..

[7] Teunissen, C. E. *et al.* Multi-omics interdisciplinary research integration to accelerate dementia biomarker development (MIRIADE). *Front. Neurol.* 1302 (2022). 10.3389/fneur.2022.890638 PMC9315267 35903119

[8] Ryan J, Fransquet P, Wrigglesworth J, Lacaze P (2018). Phenotypic heterogeneity in dementia: A challenge for epidemiology and biomarker studies. Front. Public Health.

[9] Jutten RJ (2020). Identifying and predicting heterogeneity in cognitive decline among individuals with prodromal Alzheimer’s disease using a latent class analysis: Neuropsychiatry and behavioral neurology: The neuropsychiatry of subjective cognitive disorder and prodromal ad. Alzheimer’s Dement..

[10] Bellucci A, Bugiani O, Ghetti B, Spillantini MG (2011). Presence of reactive microglia and neuroinflammatory mediators in a case of frontotemporal dementia with p301s mutation. Neurodegener. Dis..

[11] Heneka MT (2015). Neuroinflammation in Alzheimer’s disease. Lancet Neurol..

[12] Buchman AS (2016). Higher brain BDNF gene expression is associated with slower cognitive decline in older adults. Neurology.

[13] Amin J (2020). Neuroinflammation in dementia with Lewy bodies: A human post-mortem study. Transl. Psychiatry.

[14] Van Steenoven I (2020). Identification of novel cerebrospinal fluid biomarker candidates for dementia with Lewy bodies: A proteomic approach. Mol. Neurodegener..

[15] Jiang Y (2022). Large-scale plasma proteomic profiling identifies a high-performance biomarker panel for Alzheimer’s disease screening and staging. Alzheimer’s Dement..

[16] Kivisäkk P (2022). Plasma biomarkers for prognosis of cognitive decline in patients with mild cognitive impairment. Brain Commun..

[17] del Campo, M. *et al.* CSF proteome profiling across the Alzheimer’s disease spectrum reflects the multifactorial nature of the disease and identifies specific biomarker panels. *Nat. Aging* 1–14 (2022). 10.1038/s43587-022-00300-1 PMC10292920 37118088

[18] Whelan CD (2019). Multiplex proteomics identifies novel CSF and plasma biomarkers of early Alzheimer’s disease. Acta Neuropathol. Commun..

[19] Higginbotham L (2020). Integrated proteomics reveals brain-based cerebrospinal fluid biomarkers in asymptomatic and symptomatic alzheimer’s disease. Sci. Adv..

[20] Bader JM (2020). Proteome profiling in cerebrospinal fluid reveals novel biomarkers of Alzheimer’s disease. Mol. Syst. Biol..

[21] Zetterberg H, Blennow K (2021). Moving fluid biomarkers for Alzheimer’s disease from research tools to routine clinical diagnostics. Mol. Neurodegener..

[22] Martino Adami PV (2022). Matrix metalloproteinase 10 is linked to the risk of progression to dementia of the Alzheimer’s type. Brain.

[23] Teunissen CE (2016). Novel diagnostic cerebrospinal fluid biomarkers for pathologic subtypes of frontotemporal dementia identified by proteomics. Alzheimer’s Dement. Diagn. Assess. Dis. Monit..

[24] Libiger O (2021). Longitudinal CSF proteomics identifies NPTX2 as a prognostic biomarker of Alzheimer’s disease. Alzheimer’s Dement..

[25] Tijms BM (2020). Cerebrospinal fluid proteomic profiles predict progression to dementia in prodromal ad: Biomarkers (non-neuroimaging): Longitudinal and prognostic biomarker studies. Alzheimer’s Dement..

[26] Vromen EM (2022). CSF proteomic signature predicts progression to Alzheimer’s disease dementia. Alzheimer’s Dement. Transl. Res. Clin. Interv..

[27] Haytural H (2021). Insights into the changes in the proteome of Alzheimer disease elucidated by a meta-analysis. Sci. Data.

[28] Del Campo M (2015). Facilitating the validation of novel protein biomarkers for dementia: An optimal workflow for the development of sandwich immunoassays. Front. Neurol..

[29] Petrera A (2020). Multiplatform approach for plasma proteomics: complementarity of Olink proximity extension assay technology to mass spectrometry-based protein profiling. J. Proteome Res..

[30] Hok-A-Hin, Y. S., Willemse, E. A., Teunissen, C. E. & Del Campo, M. Guidelines for CSF processing and biobanking: Impact on the identification and development of optimal CSF protein biomarkers. *Cerebrospinal Fluid (CSF) Proteomics: Methods and Protocols* 27–50 (2019). 10.1007/978-1-4939-9706-0_2 31432404

[31] Proust-Lima, C., Philipps, V. & Liquet, B. Estimation of extended mixed models using latent classes and latent processes: The r package lcmm. arxiv 2015. arXiv preprint arXiv:1503.00890 (2016).

[32] de Leeuw FA (2017). Blood-based metabolic signatures in Alzheimer’s disease. Alzheimer’s Dement. Diagn. Assess. Dis. Monit..

[33] Szklarczyk D (2023). The string database in 2023: Protein–protein association networks and functional enrichment analyses for any sequenced genome of interest. Nucleic Acids Res..

[34] Tijms BM (2020). Pathophysiological subtypes of Alzheimer’s disease based on cerebrospinal fluid proteomics. Brain.

[35] Bertens D (2017). The effect of diagnostic criteria on outcome measures in preclinical and prodromal Alzheimer’s disease: Implications for trial design. Alzheimer’s Dement. Transl. Res. Clin. Interv..

[36] Wang, W.-Y., Tan, M.-S., Yu, J.-T. & Tan, L. Role of pro-inflammatory cytokines released from microglia in Alzheimer’s disease. *Ann. Transl. Med.* **3** (2015). 10.3978/j.issn.2305-5839.2015.03.49 PMC4486922 26207229

[37] Leng F, Edison P (2021). Neuroinflammation and microglial activation in Alzheimer disease: Where do we go from here?. Nat. Rev. Neurol..

[38] Cardenas V (2011). Brain atrophy associated with baseline and longitudinal measures of cognition. Neurobiol. Aging.

[39] Sutphen CL (2018). Longitudinal decreases in multiple cerebrospinal fluid biomarkers of neuronal injury in symptomatic late onset Alzheimer’s disease. Alzheimer’s Dement..

[40] Trares K (2022). Association of the inflammation-related proteome with dementia development at older age: Results from a large, prospective, population-based cohort study. Alzheimer’s Res. Ther..

[41] Dammer, E. B. *et al.* Multi-platform proteomic analysis of Alzheimer’s disease cerebrospinal fluid and plasma reveals network biomarkers associated with proteostasis and the matrisome. *bioRxiv* (2022). 10.1186/s13195-022-01113-5 PMC9670630 36384809

[42] Perna L (2022). Risk of late-onset depression and cognitive decline: Results from inflammatory proteome analyses in a prospective population-based cohort study. Am. J. Geriatr. Psychiatry.

[43] Dong Y, Dekens DW, De Deyn PP, Naudé PJ, Eisel UL (2015). Targeting of tumor necrosis factor alpha receptors as a therapeutic strategy for neurodegenerative disorders. Antibodies.

[44] Montgomery SL (2011). Ablation of TNF-RI/RII expression in Alzheimer’s disease mice leads to an unexpected enhancement of pathology: Implications for chronic pan-TNF-$$\alpha $$ suppressive therapeutic strategies in the brain. Am. J. Pathol..

[45] Probert L (2015). TNF and its receptors in the CNS: The essential, the desirable and the deleterious effects. Neuroscience.

[46] Robertson IB, Rifkin DB (2016). Regulation of the bioavailability of TGF-$$\beta $$ and TGF-$$\beta $$-related proteins. Cold Spring Harb. Perspect. Biol..

[47] Zhao B, Xu S, Dong X, Lu C, Springer TA (2018). Prodomain-growth factor swapping in the structure of pro-TGF-$$\beta $$1. J. Biol. Chem..

[48] Caraci F (2012). Dysfunction of TGF-$$\beta $$1 signaling in Alzheimer’s disease: Perspectives for neuroprotection. Cell Tissue Res..

[49] Bosco P (2013). Role of the transforming-growth-factor-$$\beta $$1 gene in late-onset Alzheimer’s disease: Implications for the treatment. Curr. Genom..

[50] Wyss-Coray T (2001). TGF-$$\beta $$1 promotes microglial amyloid-$$\beta $$ clearance and reduces plaque burden in transgenic mice. Nat. Med..

[51] Ercan-Herbst E (2019). A post-translational modification signature defines changes in soluble tau correlating with oligomerization in early stage alzheimer’s disease brain. Acta Neuropathol. Commun..

[52] Janelidze S (2020). Cerebrospinal fluid p-tau217 performs better than p-tau181 as a biomarker of Alzheimer’s disease. Nat. Commun..

[53] Kim J (2017). Tracking cognitive decline in amnestic mild cognitive impairment and early-stage Alzheimer dementia: Mini-mental state examination versus neuropsychological battery. Dement. Geriatr. Cogn. Disord..

[54] Jutten, R. J. *et al.* Why a clinical trial is as good as its outcome measure: A framework for the selection and use of cognitive outcome measures for clinical trials of Alzheimer’s disease. *Alzheimer’s Dement.* (2022). 10.1002/alz.12773 PMC9931632 36086926

[55] Mitchell AJ (2009). A meta-analysis of the accuracy of the mini-mental state examination in the detection of dementia and mild cognitive impairment. J. Psychiatr. Res..

[56] Silverberg NB (2011). Assessment of cognition in early dementia. Alzheimer’s Dement..

[57] Wang J (2016). Adcoms: A composite clinical outcome for prodromal Alzheimer’s disease trials. J. Neurol. Neurosurg. Psychiatry.

[58] Jutten RJ (2020). The cognitive-functional composite is sensitive to clinical progression in early dementia: Longitudinal findings from the catch-cog study cohort. Alzheimer’s Dement. Transl. Res. Clin. Interv..

[59] Jutten RJ (2021). Identifying sensitive measures of cognitive decline at different clinical stages of Alzheimer’s disease. J. Int. Neuropsychol. Soc..

[60] Cohen S, Cummings J, Knox S, Potashman M, Harrison J (2022). Clinical trial endpoints and their clinical meaningfulness in early stages of Alzheimer’s disease. J. Prev. Alzheimer’s Dis..

[61] Smits LL (2015). Trajectories of cognitive decline in different types of dementia. Psychol. Med..

[62] O’Caoimh R, Molloy DW (2019). Comparing the diagnostic accuracy of two cognitive screening instruments in different dementia subtypes and clinical depression. Diagnostics.

[63] Zhou X (2017). Impaired prosaposin lysosomal trafficking in frontotemporal lobar degeneration due to progranulin mutations. Nat. Commun..

[64] Moors TE (2019). Characterization of brain lysosomal activities in GBA-related and sporadic Parkinson’s disease and dementia with Lewy bodies. Mol. Neurobiol..

[65] Buuren, S. *et al.* Multivariate imputation by chained equations. *Comprehensive R Archive* **587** (2019).

[66] Lundberg, S. M. & Lee, S.-I. A unified approach to interpreting model predictions. *Adv. Neural Inf. Process. Syst.* **30** (2017).

[67] Bates, D., Mächler, M., Bolker, B. & Walker, S. Fitting linear mixed-effects models using lme4. arXiv preprint arXiv:1406.5823 (2014).

[68] Wu T (2021). clusterprofiler 4.0: A universal enrichment tool for interpreting omics data. Innovation.

