# Supplementary material for: Discovery of novel CSF biomarkers to predict progression in dementia using machine learning
Source: Sci Rep. 2023 Apr 21;13:6531. doi: 10.1038/s41598-023-33045-x (PMC10121677; doi:10.1038/s41598-023-33045-x)
Supplement: Supplementary file 1 — Supplementary Information. [file 41598_2023_33045_MOESM1_ESM.pdf]

Discovery of novel CSF biomarkers to predict progression in dementia using machine learning

Dea Gogishvili[1,⊕], Eleonora M Vromen[2,3], Sascha Koppes - den Hertog[3,4], Afina W Lemstra[2,3], Yolande A L Pijnenburg[2,3], Pieter Jelle Visser[2,3,5,6], Betty M Tijms[2,3], Marta Del Campo[4,7,8], The Alzheimer's Disease Neuroimaging Initiative[*], Sanne Abeln[1,9], Charlotte E Teunissen[3,4], and Lisa Vermunt[2,3,4]

[1]Computer Science, Vrije Universiteit Amsterdam, The Netherlands
[2]Alzheimer Center Amsterdam, Neurology, Vrije Universiteit Amsterdam, Amsterdam UMC location VUmc, Amsterdam, The Netherlands
[3]Amsterdam Neuroscience, Neurodegeneration, Amsterdam, The Netherlands
[4]Neurochemistry Laboratory, Department of Clinical Chemistry, Amsterdam Neuroscience, Vrije Universiteit Amsterdam, Amsterdam UMC, Amsterdam, The Netherlands
[5]Alzheimer Center Limburg, School for Mental Health and Neuroscience, Maastricht University, Maastricht, the Netherlands
[6]Department of Neurobiology, Care Sciences and Society, Division of Neurogeriatrics, Karolinska Institutet, Stockholm Sweden
[7]Barcelonaβeta Brain Research Center, Pasqual Maragall Foundation, Barcelona, Spain
[8]Departamento de Ciencias Farmacéuticas y de la Salud, Facultad de Farmacia, Universidad San Pablo-CEU, CEU Universities, Madrid, Spain
[9]CWI, Amsterdam, The Netherlands
[⊕]d.gogishvili@vu.nl
[*]Data used in preparation of this article were obtained from the Alzheimer's Disease Neuroimaging Initiative (ADNI) database (adni.loni.usc.edu). As such, the investigators within the ADNI contributed to the design and implementation of ADNI and/or provided data but did not participate in analysis or writing of this report. A complete listing of ADNI investigators can be found at: http://adni.loni.usc.edu/wp-content/uploads/how_to_apply/ADNI_Acknowledgement_List.pdf

ABSTRACT

Providing an accurate prognosis for individual dementia patients remains a challenge since they greatly differ in rates of cognitive decline. In this study, we used machine learning techniques with the aim to identify cerebrospinal fluid (CSF) biomarkers that predict the rate of cognitive decline within dementia patients. First, longitudinal mini-mental state examination scores (MMSE) of 210 dementia patients were used to create fast and slow progression groups. Second, we trained random forest classifiers on CSF proteomic profiles and obtained a well-performing prediction model for the progression group (ROC-AUC = 0.82). As a third step, Shapley values and Gini feature importance measures were used to interpret the model performance and identify top biomarker candidates for predicting the rate of cognitive decline. Finally, we explored the potential for each of the 20 top candidates in internal sensitivity analyses. TNFRSF4 and TGF β-1 emerged as the top markers, being lower in fast-progressing patients compared to slow-progressing patients. Proteins of which a low concentration was associated with fast progression were enriched for cell signalling and immune response pathways. None of our top markers stood out as strong individual predictors of subsequent cognitive decline. This could be explained by small effect sizes per protein and biological heterogeneity among dementia patients. Taken together, this study presents a novel progression biomarker identification framework and protein leads for personalised prediction of cognitive decline in dementia.

Supporting Information

Supplementary tables

Table S1. The list of 20 most promising biomarkers and their relevance. FI, Gini feature importance; LQ-HQ, contrast (p-value) of difference between the lowest and the highest quartiles for each biomarker, negative contrast (LQ-HQ < 0) value indicates that the lower quartile progresses faster than the highest quartile; LOD, the limit of detection (detectability); Significance is calculated using 'emtrends' function in R; all, all patients with dementia (n=210); AD, patients with AD dementia (n=119); **** 0.0001, *** 0.001, ** 0.01, * 0.05, n.s. not significant, dash (-) not measured.

Biomarker	FI	ANCOVA	SHAP	LQ-HQ contrast (all)	LQ-HQ contrast (AD)	LOD	Validation	
							ADNI[34]	Trend
CLEC1B	1	-0.25	2	-1.32**	-2.04***	78	-	-
TNFRSF4	2	-0.18*	3	-0.97*	-1.31*	100	-	-
TGF β-1	3	-0.18*	1	-1.29**	-1.64***	100	-0.05 (n.s.)	y
PLXNB1	4	-0.21**	4	-1.30**	-1.60**	100	-	-
ITGB2	5	-0.20*	>20	0.63 (n.s.)	-0.04 (n.s.)	100	-	-
MYOC	6	0.39*	>20	0.34 (n.s.)	0.53 (n.s.)	100	-	-
PVRL4	7	-0.19**	5	-1.12*	-1.88****	100	-	-
WFDC2	8	-0.11**	6	-0.94*	-1.53**	100	-	-
TNFRSF6B	9	-0.39**	8	-1.23**	-1.47*	100	-	-
ITGAM	10	0.20*	>20	1.15*	0.60 (n.s.)	28	-	-
EPHB6	11	-0.36**	10	-0.90*	-1.59**	100	-	-
GFRA2	12	-0.12**	9	-0.84*	-1.09 (n.s.)	100	-	-
STC1	13	-0.28**	16	-0.53 (n.s.)	-0.72 (n.s.)	100	-	-
MATN3	14	-0.11	11	-0.63 (n.s.)	-1.09 (n.s.)	100	-	-
ARSB	15	0.11	>20	0.44 (n.s.)	0.51 (n.s.)	100	-	-
CD48	16	-0.23*	7	-0.98 (n.s.)	-0.80 (n.s.)	100	-	-
FLRT2	17	-0.17*	13	-0.79 (n.s.)	-0.99 (n.s.)	100	-	-
ADAM 23	18	-0.12**	>20	-0.70 (n.s.)	-1.32*	100	-	-
β-NGF-1	19	-0.16**	15	-0.98*	-1.72**	100	-0.50 (n.s.)	y
SPON1	20	0.09	>20	0.15 (n.s.)	0.28 (n.s.)	78	-2.11*	n

Supplementary figures

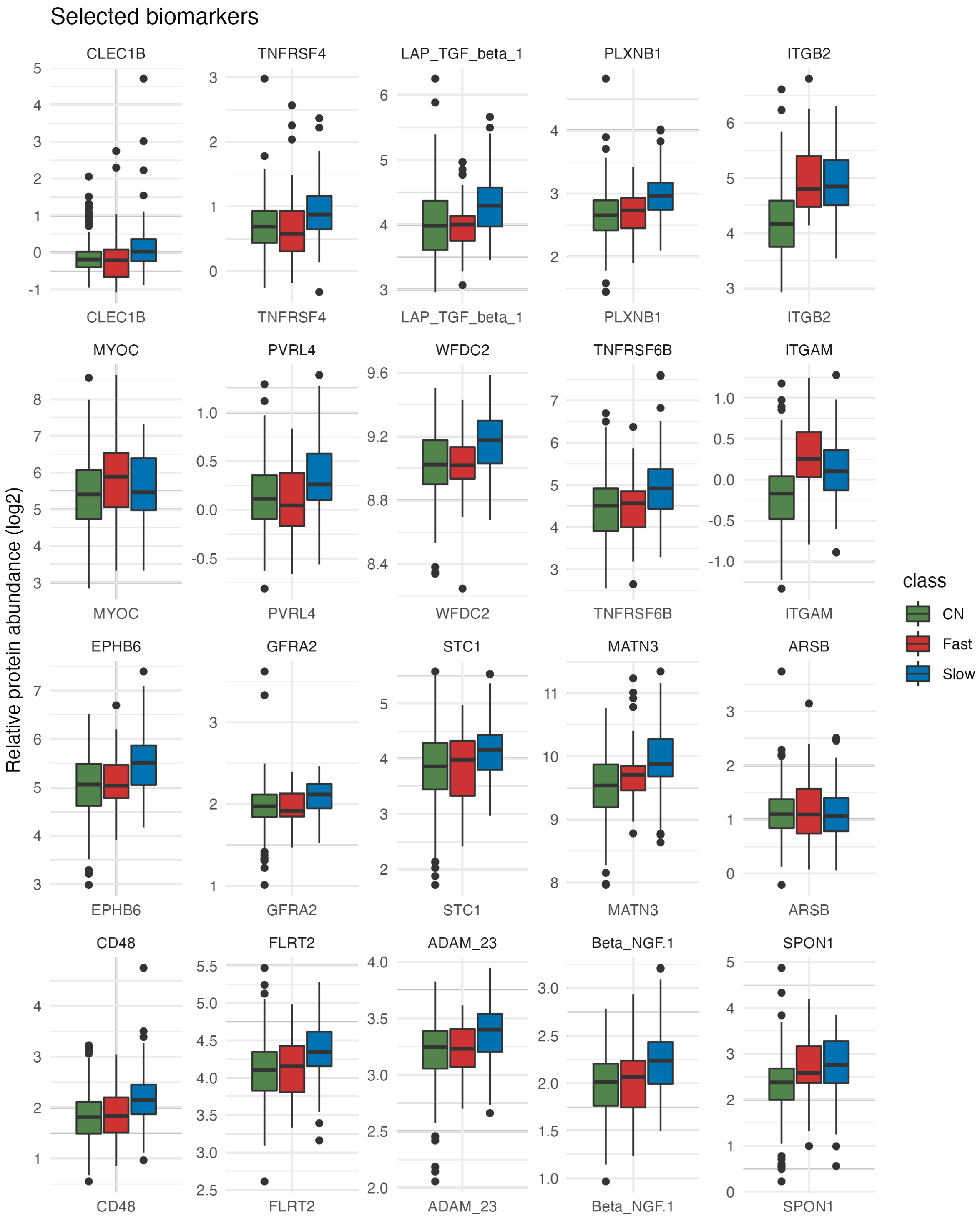

Figure S1. Normalised protein expression (NPX) values of selected biomarkers. Relative protein abundance (log2) of the top 20 biomarkers with the highest feature importance based on the best-performing machine learning model. CN, cognitively normal.

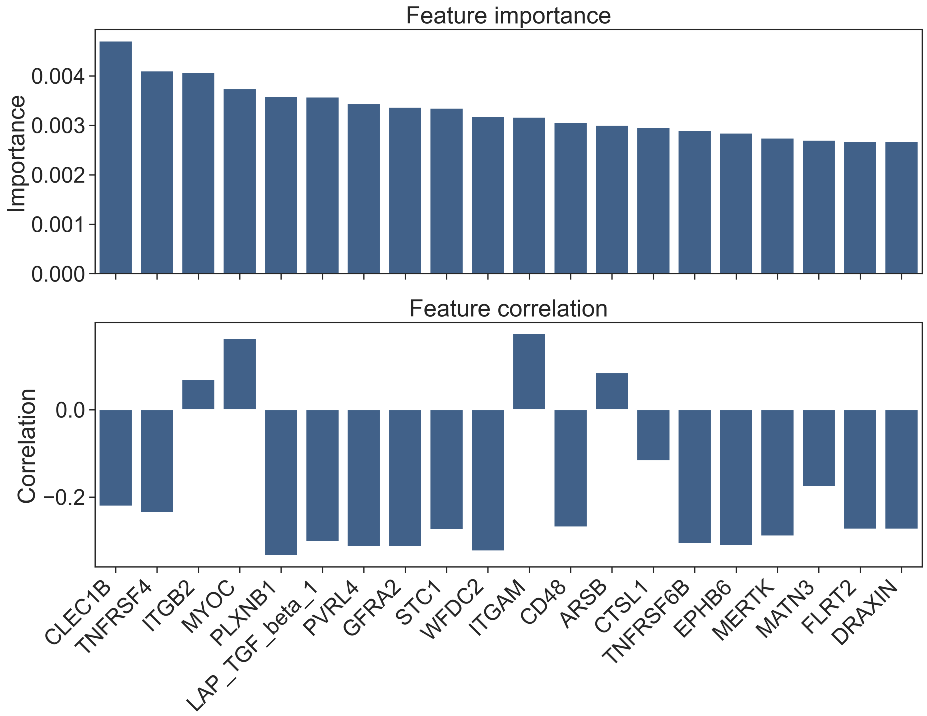

Figure S2. Gini feature importance and feature correlation analysis for the Olink + age model. The barplot on top of the graph shows the top 20 most important features for predicting the rate of decline. The barplot below shows the correlation with the progression. The top 17 out of 20 biomarkers overlap with the feature importance ranking of the Olink model, which was trained on protein measurements only.

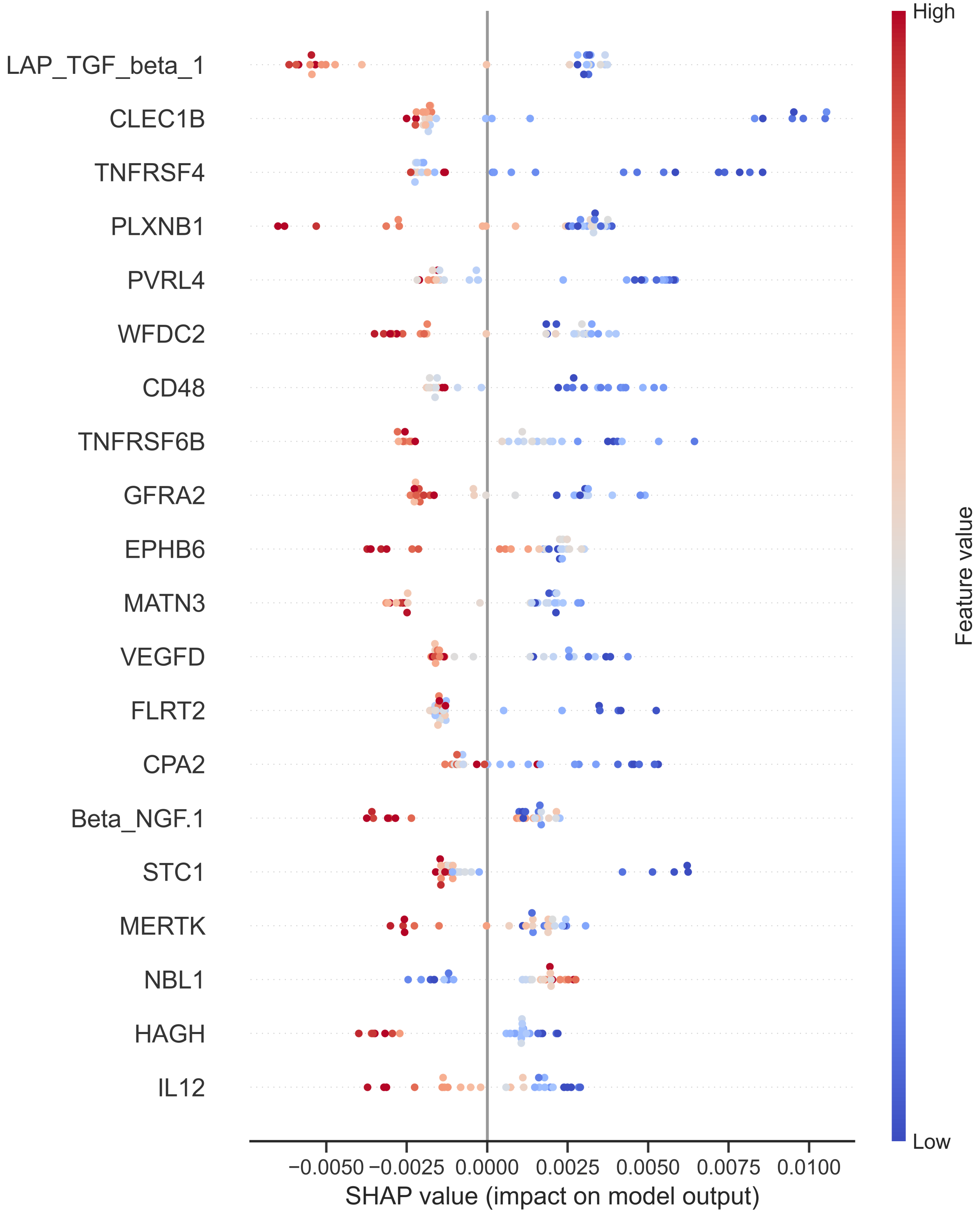

Figure S3. Summary SHAP plot shows a global level in which top features contribute to the prediction. Biomarkers (features) are ranked in descending order. Each dot represents an instance (a patient) in our training data. The horizontal location shows whether the effect of that value is associated with a higher (fast) or lower (slow) prediction. The color palette shows the value of each feature for each case being high or low for the respective observation. In the case of LAP TGF β-1 higher expression values (red) are associated with the slowly progressing patients and lower levels (blue) are linked to fast-progressing patients.

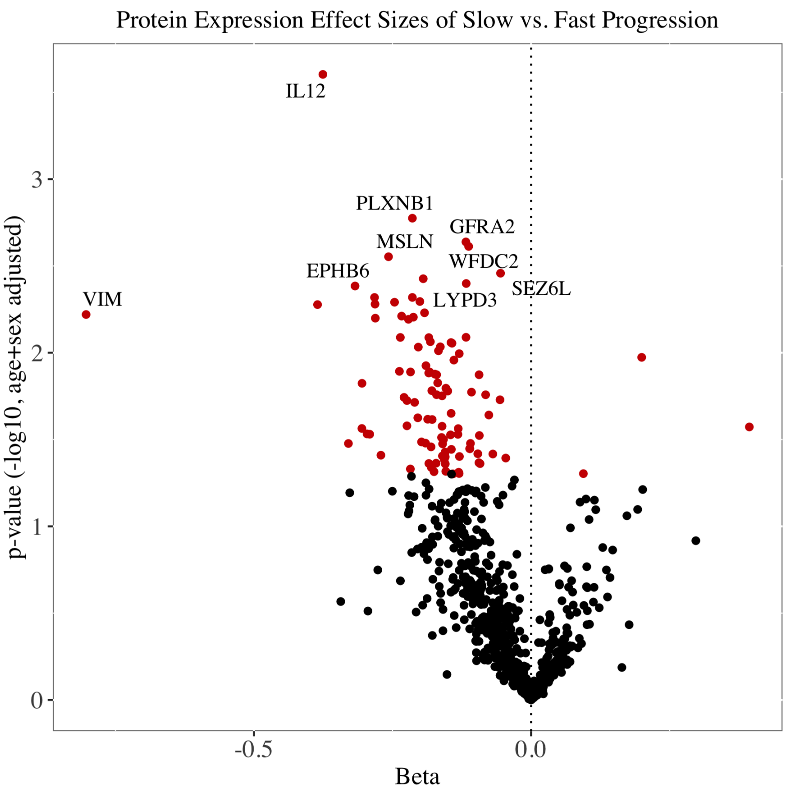

Figure S4. ANCOVA results adjusted for age and sex Dots coloured in red are proteins significantly different between progression groups (p-value < 0.05).

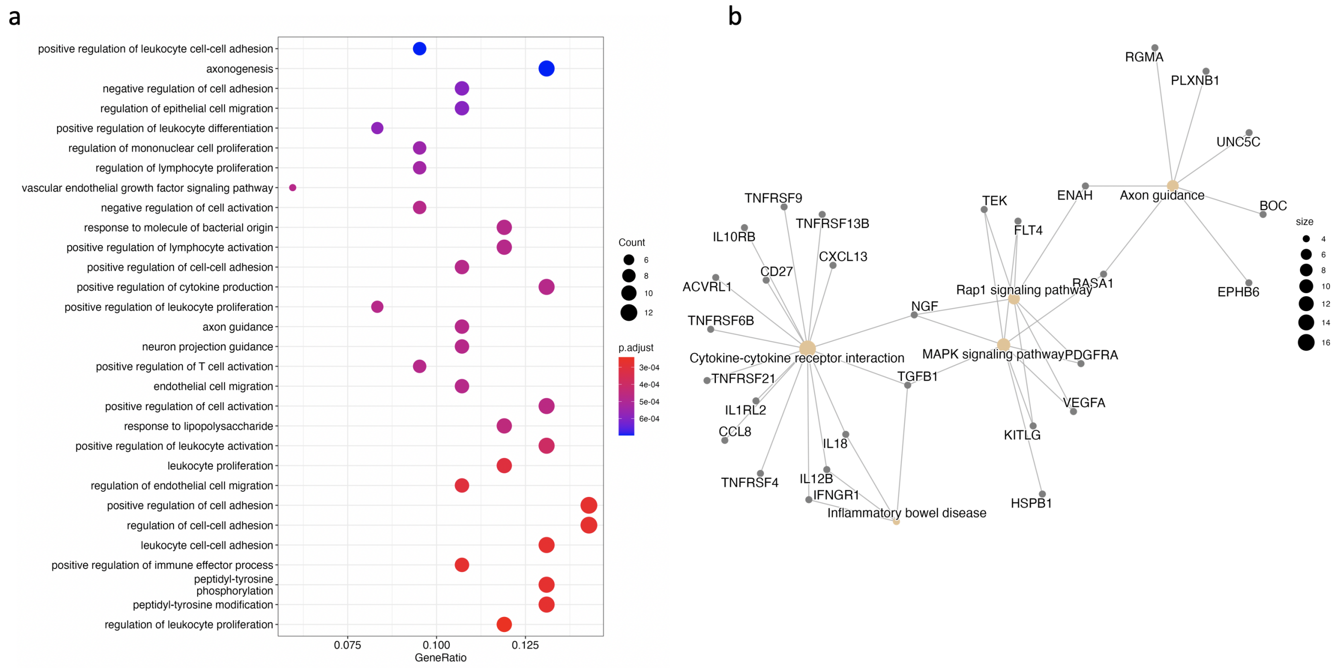

Figure S5. Visualisation of GO terms and KEGG pathway enrichment results without defined background. (a) Dot plot depicts significantly enriched GO terms, p-value scores (coloured), gene ratio, count (dot size). **(b)** Visualises the connection of enriched KEGG pathways. Nodes represent processes and edges represent a set of proteins selected based on machine learning and ANCOVA results.

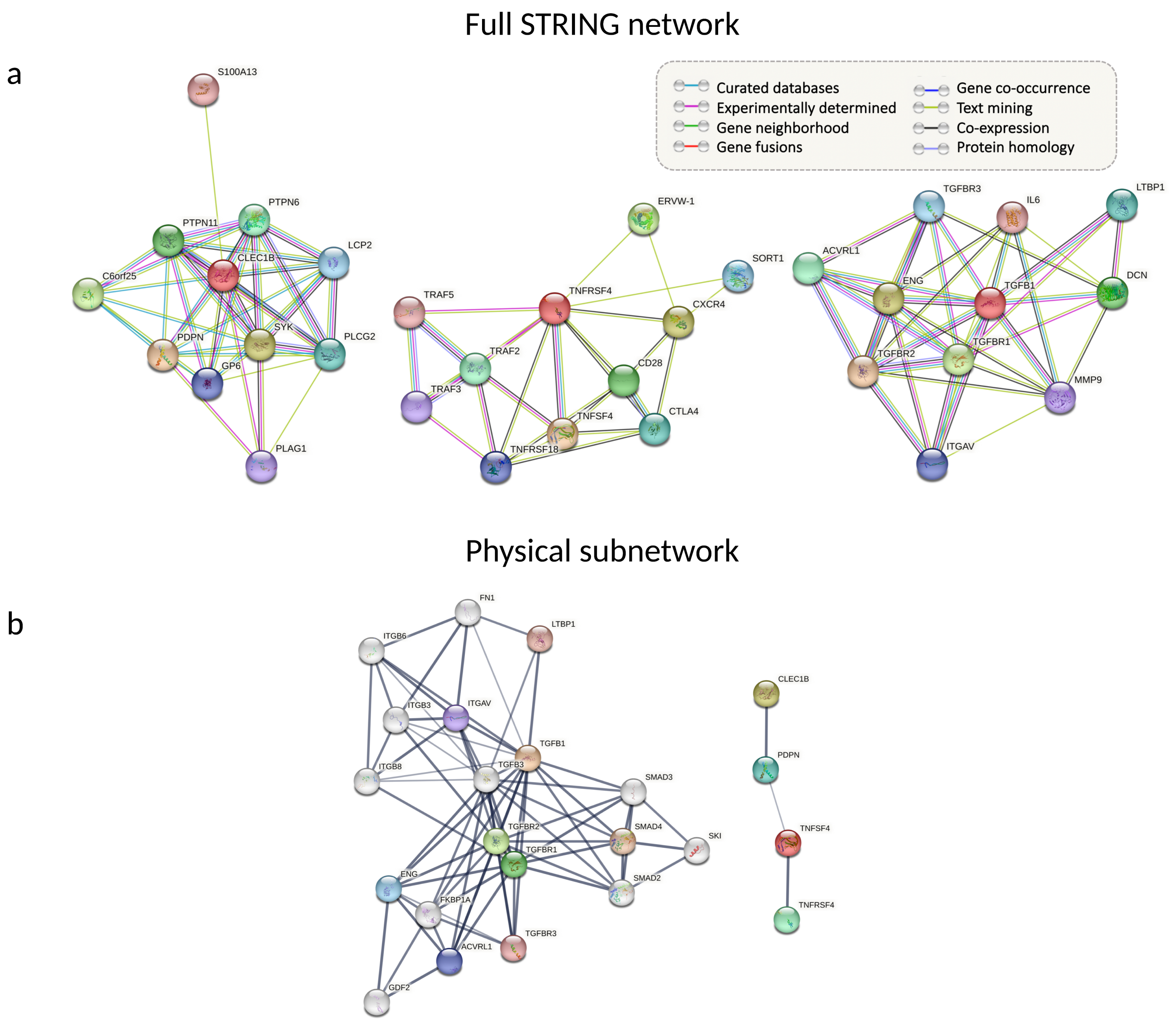

Figure S6. Protein-protein interaction networks were constructed using a graph-based approach using STRING. **(a)** Full STRING network (both functional and physical protein associations). Each node in the network represents a single protein. Edges between nodes indicate a specific protein-protein interaction. **(b)** Physical subnetwork CLEC1B, TNFRSF4, and TGF Beta-1. The edges indicate that the proteins are part of a physical complex and the line thickness indicates the strength of data supporting the interaction.
